# A Bibliometric Analysis of Global Research Hotspots and Progress on Microbial Extracellular Polymeric Substances in Bioremediation

**DOI:** 10.3390/microorganisms14061218

**Published:** 2026-05-27

**Authors:** Su Yan, Shiqi Xue, Xinting Lv, Jiaxin Li, Ningxuan Ma, Manning Wang, Yue Quan

**Affiliations:** 1 Department of Agricultural Resources and Environment, Yanbian University, Yanji 133002, China; 15843615021@163.com (S.Y.); 15545240805@163.com (X.L.); jxjx020204@163.com (J.L.); 2 Department of Geography and Ocean Sciences, Yanbian University, Hunchun 133300, China; 3 Jilin Black Soil Protection Monitoring Center, Jilin 132000, China; wangmanning2026@163.com

**Keywords:** extracellular polymeric substances, bioremediation, bibliometrics, emerging pollutants

## Abstract

Extracellular polymeric substances (EPSs) are high-molecular-weight biopolymers secreted by microorganisms, showing great potential for bioremediation. However, comprehensive analyses of the development context and quantitative research on the overall trends of EPSs in bioremediation are lacking. This study conducted a systematic bibliometric analysis of microbial EPS research using VOSviewer and CiteSpace. Keyword burst and thematic evolution analysis indicate a distinct thematic shift: early research focused on “structural characterization and adsorption mechanisms of EPSs”, whereas current hotspots highlight interactions with emerging pollutants (e.g., microplastics, antibiotics, and antibiotic resistance genes (ARGs)). EPSs significantly influence the environmental fate and removal efficiency of emerging pollutants through multiple pathways, including physical adsorption, chemical complexation, photocatalytic degradation, and electron transfer. For microplastic remediation, EPSs mediate hetero-aggregation, surface modification, and biodegradation processes. In antibiotic removal, EPSs function through biosorption, biodegradation, and photosensitized degradation. Regarding the mitigation of ARGs, EPSs can either suppress or facilitate their horizontal gene transfer, depending on their composition and environmental conditions. Additionally, as electroactive medium, EPSs play a crucial role in facilitating electron transfer, enhancing nitrogen removal, and promoting heavy metals reduction. This study systematically reviewed the current status and research hotspots of EPSs in bioremediation. However, practical applicability remains constrained by challenges such as low production yield and high costs. Future directions to address these limitations are also outlined to guide further development.

## 1. Introduction

Environmental pollution has emerged as one of the most pressing global challenges of our era with the acceleration of globalization and industrialization. With a substantial volume of pollutants being released into the environment, bioremediation has gained increasing attention as a promising and sustainable solution due to its low cost and operational simplicity [1]. Microbial bioremediation is widely recognized for its dependence on natural processes. It is widely applied for completely mineralizing organic pollutants into CO_2_ and H_2_O rather than merely transferring pollutants between different forms or media, owing to its energy efficiency, eco-friendliness, and low operational costs in both in situ and ex situ scenarios [2]. This process avoids secondary pollution and achieves thorough remediation of contaminated environments. Microorganisms achieve the degradation, transformation, or immobilization of pollutants through their metabolic activities, making microbial bioremediation the most widely used approach among remediation technologies [3]. Previous studies mainly focused on three aspects: the functional microbial consortia themselves, pollutant removal efficiency, and environmental factor regulation. However, systematic research on microbial extracellular polymeric substances (EPSs) remains lacking.

Microorganisms including prokaryotes and eukaryotes are induced to produce EPSs under certain conditions [4]. EPSs exhibit multiple functions, including adsorption, flocculation, protection, degradation, and information transfer, thereby playing a crucial role in microbial growth and metabolism, environmental pollution, and biogeochemical cycles [5,6]. EPSs, as complex high-molecular-weight biopolymers mixtures, are primarily composed of polysaccharides and proteins (70–80%), with nucleic acids, lipids, and humic substances constituting the remaining portion [7,8]. Together, these components form a protective matrix that encapsulates microbial cells. The structures and compositions of EPSs are not fixed, but are significantly regulated by several factors such as microbial community structure, metabolic status, and multiple environmental factors including nutrients (carbon, nitrogen, and phosphorus sources), temperature, pH, salinity, and pollutants [9,10]. Additionally, EPSs contain various functional groups, including carboxyl groups (-COOH), amino groups (-NH_2_), and phosphate groups (-PO_4_^3−^) [11]. The complex network structure and various functional groups of EPSs can interact with pollutants through mechanisms such as electrostatic adsorption, complexation, ion exchange, and hydrophobic interactions. These processes collectively facilitate contaminant immobilization, transformation, and degradation [12].

Based on their solubility and binding affinity to microbial cells, EPSs are primarily classified as soluble EPSs (S-EPSs), loosely bound EPSs (LB-EPSs), and tightly bound EPSs (TB-EPSs) [13]. The three types of EPS exhibit differences in chemical composition and physicochemical properties, which can lead to distinct behaviors in certain systems. For example, S-EPSs, LB-EPSs and TB-EPSs were successfully extracted from *Pseudomonas aeruginosa*, and the S-EPSs mainly composed of humic acids can form a passive film to inhibit corrosion on EH40 steel in 3.5 wt% NaCl solution, while LB-EPSs and TB-EPSs mainly composed of proteins show corrosion acceleration effects [14]. S-EPSs inhibited corrosion and TB-EPSs promoted corrosion on EH40 steel from sulfate-ho6reducting bacteria under anaerobic conditions. This opposite effect is attributed to their compositional and spatial differences: S-EPSs, which are primarily composed of humic acids and located in the outermost layer, adhere to the steel surface to form a protective film. In contrast, TB-EPSs, which are protein-based and tightly bound to the cell wall, possess active groups (such as C=O and P=O) that exhibit strong complexation ability with Fe^2+^ [15]. However, such clear functional differentiation among EPS fractions has rarely been investigated in the context of pollutant remediation, where most studies have used total EPS extracts.

EPSs exhibit the potential functions to interact with diverse pollutants, and play a crucial role in regulating the migration, transformation, and fate of pollutants [16]. The functions of EPSs are closely related to the type of remediation system (activated sludge, biofilm), metabolic environment (aerobic, anaerobic), and microbial community structure (bacteria, fungi, single-strain, mixed-strain) [11]. Significant differences exist in the production mechanisms, compositional characteristics, and remediation functions of EPSs across different systems, which directly affect the efficiency and stability of bioremediation.

Microbial EPSs have become a key research focus in bioremediation and are used in soil remediation, soil erosion control, and wastewater treatment [17,18]. However, current research is largely confined to the fundamental properties of EPS and its role in conventional pollutant removal. A systematic, quantitative review tracing the intellectual trajectory of this field has yet to be conducted. Furthermore, existing reviews have primarily focused on the remediation of traditional contaminants. Discussions concerning emerging pollutants, such as microplastics, antibiotics, and antibiotic resistance genes (ARGs), remain fragmented. A systematic integration of the underlying mechanisms and a synthesis of overarching principles are still lacking. Bibliometrics as an analytical method based on statistics and mathematics enables objective, rapid, accurate, and intuitive analysis of research progress, trends, and hotspots across various fields [19]. It provides an effective method for systematically organizing the research system of EPSs in bioremediation.

This study statistically and analytically analyzed the literature on the microbial EPSs in bioremediation based on the Web of Science (WOS) Core Collection database using VOSviewer (1.6.20) and CiteSpace (6.1.R6). This study centered on the core issue of research hotspots and future directions for microbial EPSs in bioremediation. Its contributions were threefold, distinguishing it from existing reviews. First, it filled a systemic gap in the literature by focusing on the interaction mechanisms between EPSs and emerging pollutants, particularly concerning microplastics and the dissemination of ARGs. Second, through keyword co-occurrence clustering and burst analysis, it objectively delineated current research hotspots and projects future developmental trajectories in the field. Third, this review critically examined the inherent limitations and practical challenges confronting EPS applications, thereby furnishing a scientific foundation and reference framework for subsequent investigations.

## 2. Methodology

### 2.1. Data Sources and Retrieval

**Figure 1:**
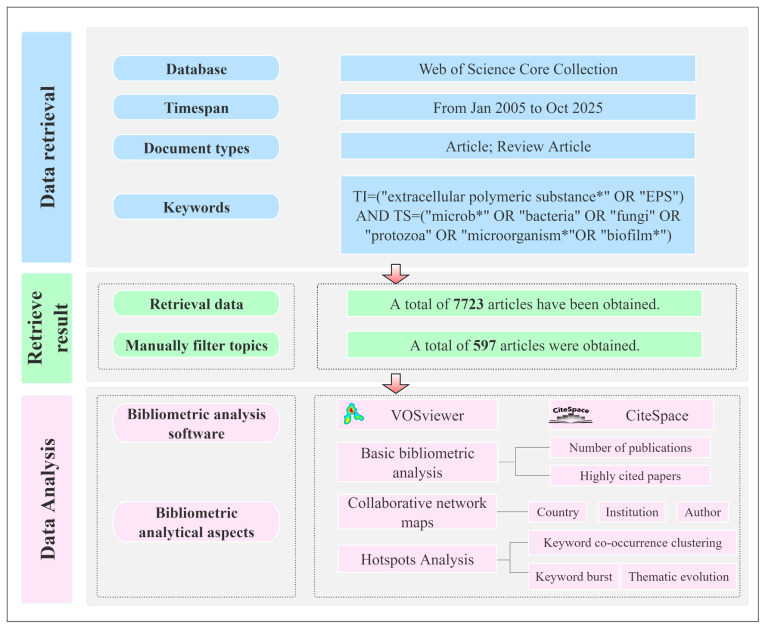
Flowchart of bibliometric methodology. Note: The * is a truncation (wildcard) operator used in the Web of Science search syntax. It matches any ending of a word root, allowing the search to capture all relevant variants (e.g., singular/plural forms, different suffixes) without listing each term explicitly.

The flowchart of this study is shown in Figure 1. This study focuses on research concerning the application of microbial EPSs in bioremediation from 2005 to 2025, with data sourced from the core academic database: WOS. Indexing was limited to the Science Citation Index Expanded (SCIE) version of the WOS database and the Social Sciences Citation Index (SSCI). WOS serves as a core repository for global academic data, encompassing information from numerous prestigious and high-impact scholarly journals worldwide [20]. It covers a wide period, and the literature collection is comprehensive and systematic. Equipped with its high-quality articles and strict screening criteria, WOS is widely regarded as the most authoritative multidisciplinary database for academic research [21].

The search keywords used were TI = (“extracellular polymeric substance*” OR “EPS”) AND TS = (“microb*” OR “bacteria” OR “fungi” OR “protozoa” OR “microorganism*”OR “biofilm*”). The search period was set from January 2005 to October 2025, and the selected document types were limited to Article and Review Article, excluding commentaries, conference proceedings, book chapters, and other irrelevant publication types. The literature retrieved from the initial search was manually screened. Two researchers independently read the titles and abstracts. Notably, “EPS” has semantic ambiguities (e.g., expanded polystyrene, electrostatic precipitator) that caused irrelevant initial records. We established clear screening criteria:

Inclusion: Studies on microbial EPSs (polysaccharides/proteins/nucleic acids/lipids) applied in bioremediation (water/soil/sediment/groundwater).

Exclusion: Non-microbial EPSs, non-bioremediation applications, unavailable full texts.

**Figure 2:**
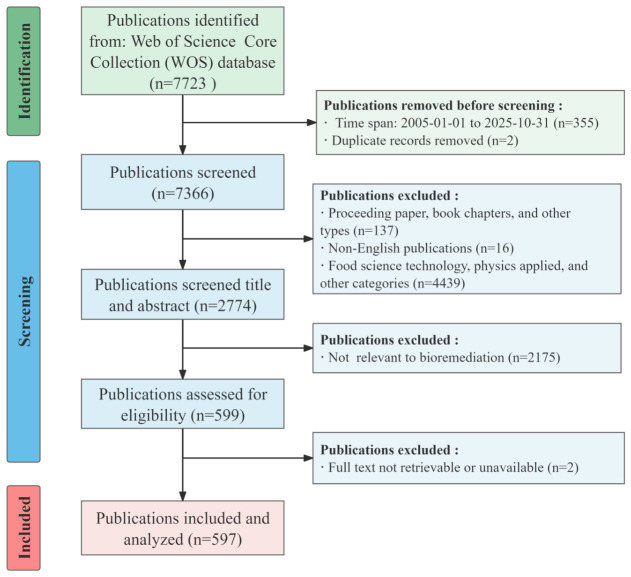
PRISMA flow diagram. Note: *n* represents the number of publications.

Any discrepancies in screening decisions were resolved through group discussion to ensure the accuracy of the final dataset. After manual screening, 597 valid articles were ultimately obtained as the data for analysis. The detailed screening and selection process is shown in the PRISMA flow diagram (Figure 2). The final set of records was exported in plain text format as “full records and cited references,” and the downloaded files were renamed to a format that the analysis software could recognize as the basis for subsequent data analysis.

Although the search strategy and screening process were rigorously optimized, this study used only the WOS database as the data source, which may lead to the underestimation or neglect of certain aspects or country-specific contributions. Future research should integrate a broader range of scientific literature and databases (such as PubMed, Scopus, Google Scholar, etc.) to enhance the comprehensiveness and depth of the analysis. Furthermore, to enhance the accuracy of keyword co-occurrence analysis, we performed data cleaning steps such as merging synonyms, standardizing singular and plural forms, and unifying abbreviations with their full terms. However, the assignment of keywords in different publications remains highly subjective, which may cause some bias in the co-occurrence analysis results. Future studies could incorporate supplementary validation using high-frequency core terms extracted from article titles and abstracts.

### 2.2. Data Analysis Methods

The bibliometric visualization analysis of the literature on microbial EPSs from the WOS database was performed using the software tools CiteSpace (6.1.R6) and VOSviewer (1.6.20). CiteSpace (6.1.R6), developed by American scholar Chaomei Chen, is a Java-based software tool for scientometric and bibliometric analysis [22]. Through techniques such as co-citation analysis, burst detection, collaborative network analysis, and timeline visualization, this software enables systematic analysis and visual representation of the knowledge structure, evolutionary patterns of research domains, and scientific collaboration networks within academic literature [23]. VOSviewer (1.6.20) is a software tool specifically developed for constructing and visualizing bibliometric networks by researchers at Leiden University in The Netherlands in 2007 [24,25].

In this study, publication volume, keyword burst analysis and trends of highly cited works were analyzed using CiteSpace (6.1.R6). VOSviewer (1.6.20) was employed to construct collaborative network maps (including co-authorship networks of country, institution, and author) and keyword co-occurrence clustering maps. The thematic evolution of relevant research was further explored via the “networkD3” package based on R (version 4.6.0).

## 3. Results

### 3.1. Number of Published Articles Analysis

**Figure 3:**
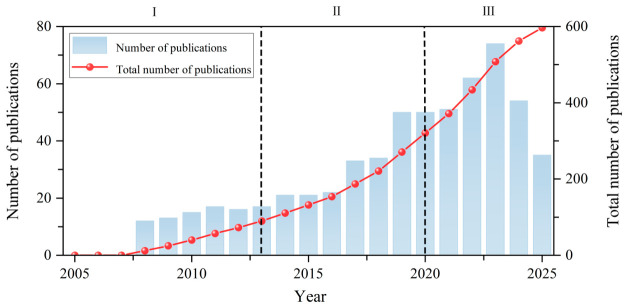
Annual publications from 2005 to 2025.

The number of published papers can reflect the development trend of a certain research field to a certain extent. Figure 3 shows the number of articles published per year on microbial EPSs. The number of publications has exhibited an overall upward trend over the past two decades. As illustrated in Figure 3, the evolution of microbial EPS research can be divided into three distinct phases. In the first stage (2005–2013), the annual publication output remained relatively low, with a total of 90 articles published over this nine-year period, averaging 10 papers per year. This indicates that EPS research was still in its nascent stage. During this stage, the core research paradigm of environmental biotechnology remained centered on the screening of functional microorganisms and the optimization of remediation process parameters. The majority of researchers regarded EPSs merely as byproducts of microbial metabolism rather than as core functional media in bioremediation. Consequently, early studies primarily focused on the extraction methods and physicochemical characterization of EPSs, laying a foundation for subsequent applications in wastewater treatment. The second stage (2014–2020), publication numbers showed a slow initial increase, followed by a substantial surge in publications, averaging 33 articles per year, representing a threefold increase compared to the earlier period. This growth was driven by two key breakthroughs specific to the field of EPS research. First, the academic community progressively recognized the central role of EPSs in the formation and functional maintenance of microbial aggregates (including activated sludge flocs, granular sludge, and biofilms) within wastewater treatment systems. Second, the adsorption and complexation capabilities of EPSs toward conventional pollutants, particularly heavy metals, were preliminarily validated. These breakthroughs facilitated the growing international academic interest in microbial EPS research, attracting an expanding number of researchers to the field. The third stage (2021–2025) represented a period of accelerated growth, with an average annual output exceeding 55 publications and a peak of 74 papers reached in 2023. This surge was likely driven by policy frameworks such as global “Dual Carbon” goals and China’s 14th Five-Year Plan for Ecological and Environmental Protection. Meanwhile, emerging global pollution challenges, such as microplastic contamination, antibiotic pollution, and the spread of ARGs, have continued to intensify, imposing higher demands on the precision and sustainability of bioremediation technologies. Owing to their multifunctional properties, including adsorption, complexation, electron transfer, and photocatalysis, EPSs have demonstrated unique advantages in the remediation of complex emerging pollutants, thereby propelling EPS-related applied research into a prominent focus in environmental remediation.

### 3.2. Analysis of Journals

**Figure 4:**
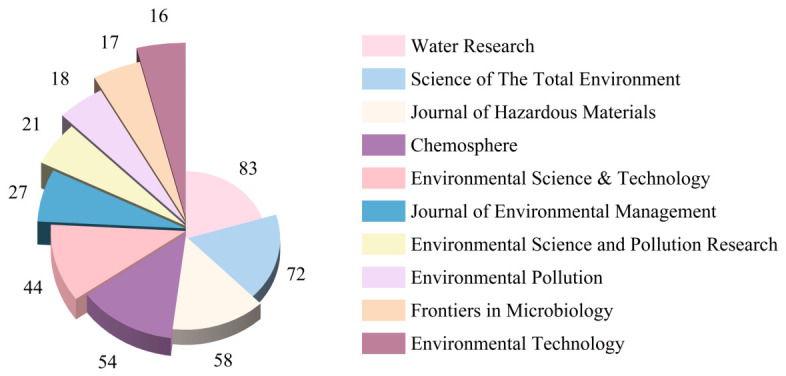
Top 10 journals with the most publications in EPS research.

Analyzing the influence of journals within a specific field allows readers to quickly identify core sources of knowledge, providing references for selecting research topics, determining submission targets, and engaging in academic exchange. A total of 83 journals have published articles on microbial EPSs. Figure 4 lists the top 10 among them, with Water Research ranking first as the leading core journal by publishing 83 articles. This dominance may stem from the strong alignment between the core functions of microbial EPSs (adsorption, flocculation, sludge regulation) and the key research needs covered by Water Research (sludge optimization, contaminant removal). Science of the Total Environment (72 articles) and Journal of Hazardous Materials (58 articles) followed in second and third place. Collectively, the top three journals accounted for 35.7% of all publications, demonstrating a strong concentration of EPS bioremediation research in leading environmental science journals. Additionally, the top 10 journals primarily covered two research areas: environmental science and engineering and microbiology, reflecting the highly interdisciplinary nature of research in this field.

### 3.3. Analysis of Countries and Institutions

**Figure 5:**
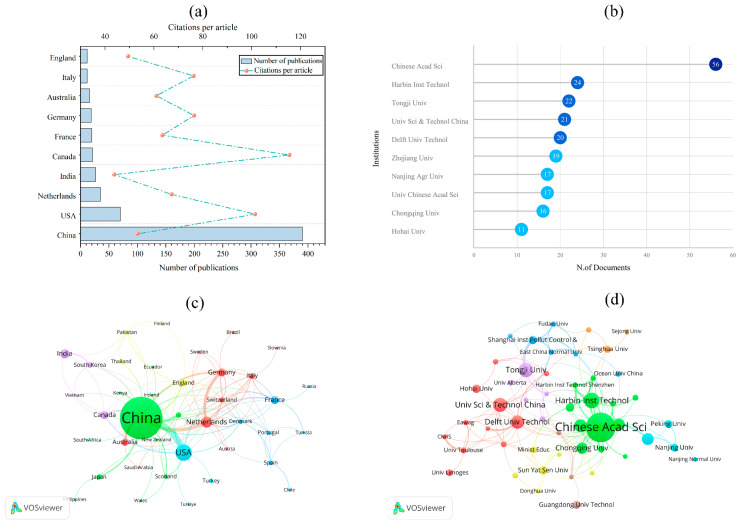
(**a**) The top 10 countries for global publications. (**b**) International country co-authorship network. (**c**) The top 10 institutions for global publications. (**d**) Institutions co-authorship network. Note: raw publication counts are influenced by factors such as database coverage and national research funding levels, which may partly explain the large difference between China and other countries.

A total of 1356 institutions from 119 countries have contributed to the literature on microbial EPSs. Figure 5a shows the distribution of publications among major contributing countries. China ranked first with 390 research articles, followed by the United States with 70 publications. The Netherlands, India, and Canada ranked third to fifth, with 35, 27, and 21 publications, respectively. This distribution may reflect various factors, including global demands for environmental governance, levels of research investment, and the development of biotechnology infrastructure. Specifically, China’s leading position is closely linked to the enormous demand for water and soil remediation arising from its rapid industrialization and urbanization, particularly driven by national strategic priorities such as black soil conservation and remediation of heavy-metal-contaminated farmland. Consequently, Chinese research has largely focused on the macroscopic performance regulation of EPSs in complex engineering systems. In contrast, research in the United States is more deeply rooted in its strong foundation in microbiology, with emphasis on elucidating the microscopic mechanisms by which EPSs govern contaminant interfacial behavior, cell protection, and interspecies electron transfer. The international collaboration network indicates that China has established cooperative relationships with several countries, including the United States, The Netherlands, and Australia, with particularly notable collaboration with the United States (Figure 5b). In terms of average citations per article, Canada led with 115.43 citations per publication, followed by the United States (101.44) and Germany (76.37). The relationship between publication volume and citation frequency across countries may offer insights into different research characteristics and the potential value of cross-regional collaboration in fostering innovation. For future research, continued attention to emerging trends in microbial EPSs and interdisciplinary collaboration could be beneficial for further advancing the field.

In addition, nine of the top ten institutions by publication volume are from China, while the other is from The Netherlands. The Chinese Academy of Sciences led with 56 publications, followed by the Harbin Institute of Technology (24) and Tongji University (22) (Figure 5c). This result demonstrates the extensive involvement and leading role of Chinese universities and research institutions in this field. Figure 5d shows the Chinese Academy of Sciences serving as a central hub in the network, with dense connections to institutions such as the Harbin Institute of Technology, Chongqing University, and Nanjing Agricultural University, highlighting its pivotal role in collaboration. Notably, the network exhibits strong regional clustering, reflecting close collaboration among Chinese institutions but limited active partnerships with leading international research organizations. Therefore, advancing cross-institutional collaboration in microbial EPS research should be prioritized to strengthen global academic exchange.

### 3.4. Author Cooperation and Publications

**Figure 6:**
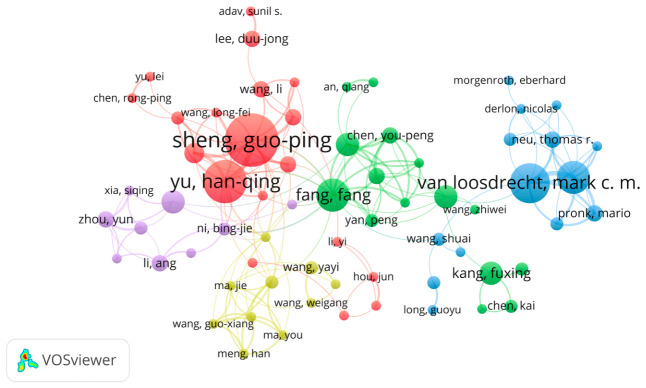
The authors co-authorship network.

A total of 2749 scholars worldwide participated in research on microbial EPSs from 2005 to 2025. Table 1 lists the top 10 authors ranked by publication output. The most prolific author was Sheng Guoping from the University of Science and Technology of China, with 16 articles. His research primarily focused on biological wastewater treatment and resource recovery. Closely following were Chinese scholars such as Yu Hanqing and Fang Fang, with 13 and 10 publications, respectively. Among international researchers, Van Loosdrecht, Mark C.M. from The Netherlands ranked highest with 12 articles. Chinese scholars accounted for 70% of the top 10 core authors, highlighting their substantial participation and leadership in microbial EPS research. The author collaboration network reveals that two teams with the largest nodes in the author cooperation network are Sheng Guoping and Van Loosdrecht, Mark C.M., indicating that they have published the most articles, contributed significantly to microbial EPS research, and laid the foundation for this field (Figure 6). Scholars from other countries also contributed to and collaborated in this field, yet most of these studies are similarly limited to research teams from the same countries. To better promote interdisciplinary integration across different research directions in microbial EPS studies, it is essential to encourage deeper collaboration between Chinese and international scholars, particularly among highly productive authors.

Furthermore, the three most influential publications in this field demonstrate the potential of microbial EPSs as environmentally benign materials for remediating contaminants in water and soil (Table 2). For example, a review published by Sheng’s team in 2010 systematically detailed the definition, extraction, characterization, production, and functions of microbial aggregates in biological wastewater treatment systems [8]. This review summarizes the key advances from the early stage of EPS research in wastewater treatment, indicating that a foundational system had been established and highlighting promising directions for the field. Additionally, this review is the first to comprehensively evaluate the extraction efficiency and degree of cell lysis of various extraction methods, thereby providing a comparable methodological framework for subsequent researchers. It is the establishment of this standardized system that enables the cross-referencing and accumulation of EPS research findings from different laboratories, accelerating the field’s transition from empirical observation to mechanistic exploration. These contributions lay essential groundwork for subsequent mechanistic exploration and engineering applications of EPSs. Costa et al. focused on the ecological functions of microbial EPSs and their regulatory role in soil aggregation, representing a study of significant academic importance [26]. The core contribution of this review lies in establishing a direct link between the physicochemical properties of EPSs (such as adhesiveness and water-holding capacity) and the ecosystem-level processes of soil aggregate formation and stabilization, thereby extending EPS research from wastewater treatment engineering into the field of soil ecology, which also accounts for its high citation rate. It is the first to systematically elucidate the mechanisms by which EPSs promote soil microaggregate formation through the adsorption of mineral particles, bridging with metal ions, and the formation of organo-mineral complexes, providing a theoretical cornerstone for soil carbon cycling regulation and erosion control. More et al. integrated research on the production, extraction, characteristics, and environmental applications of bacterial EPSs [27]. Their review marks a new phase for the systematic application of EPSs in bioremediation. This paper uniquely bridges the regulatory mechanisms of EPS biosynthesis in fundamental microbiology with practical applications in environmental engineering, such as flocculation, dewatering, and biosorption, and for the first time clearly articulates the engineering vision of employing EPSs as bioflocculants to replace conventional chemical polymers. It further systematically evaluates the application potential of EPSs in multiple scenarios, including heavy metal removal, dye decolorization, and toxic organic degradation, directly stimulating a wave of research centered on EPS-based functional materials for environmental remediation around 2014. Meanwhile, with the depth of research, scholars have proposed various mechanisms to explain the pollutant remediation effects of EPSs, ranging from ion bridging and charge neutralization in flocculation processes to functional group complexation and hydrophobic interactions in biosorption [28,29]. As environmentally benign biopolymers, the application of EPSs in wastewater treatment, sludge dewatering, and soil remediation represents a significant research direction in environmental engineering. It is anticipated that the studies in this direction will expand further, focusing on cost-effective production, large-scale implementation, and deeper mechanistic understanding.

### 3.5. Analysis of Keywords

Keywords provide a concise summary of important information in a paper [37]. Analyzing keyword co-occurrence networks can reveal developmental trends in a field, thereby supporting the effective identification of emerging topics and cutting-edge research directions. A total of 1496 keywords were contained in the literature in the field of microbial EPSs, from which 102 keywords with the highest frequency were extracted by VOSviewer (1.6.20).

#### 3.5.1. Keyword Clustering Analysis

**Figure 7:**
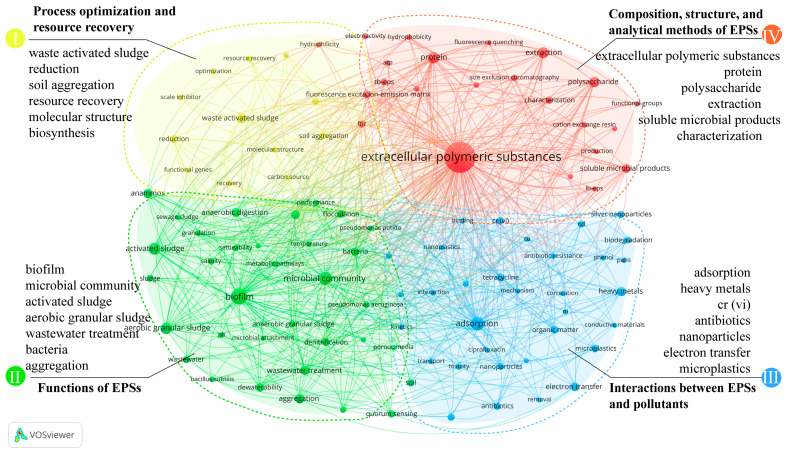
The co-occurrence of the keywords.

The keywords were clustered into four different colors based on their co-occurrence relationships (Figure 7).

Cluster 1 (yellow): Process optimization and resource recovery of EPSs

This cluster centers on the keywords “waste activated sludge”, “resource recovery”, “optimization”, “carbon source”, “functional genes”, “scale inhibitor”, etc. It reflects the research on the engineering application regulation of EPSs and corresponds to the mechanistic role of EPSs in the performance optimization of bioremediation systems and waste resource recycling. In this research dimension, EPSs are no longer just byproducts of microbial metabolism, but high-value functional products that can be recovered from waste biomass (such as waste activated sludge). Studies in this cluster focus on regulating the synthesis and secretion of EPSs through gene editing and process optimization, so as to enhance the stability of bioremediation systems (such as improving sludge sedimentation and dewatering performance) and realize the dual goals of pollutant removal and waste valorization. This cluster is an important bridge for the translation of EPS basic research into practical engineering applications.

Cluster 2 (green): Functions of EPSs in microbial aggregates and environmental systems

This cluster is dominated by keywords such as “activated sludge”, “biofilm”, “anaerobic granular sludge”, “adhesion”, “aggregation”, “quorum sensing”, “microbial community”, “denitrification”, “anammox”. It corresponds to the core basic mechanism of EPSs in bioremediation: microbial protection, community structure regulation, and mass transfer process regulation. EPSs are the core components that form the three-dimensional structure of biofilms and granular sludge. On the one hand, they build a protective barrier for microbial cells against the toxicity of pollutants and harsh environmental conditions, maintaining the stability of functional microbial communities; on the other hand, they regulate the adhesion, aggregation, and quorum sensing behavior of microorganisms, and affect the mass transfer process of pollutants, nutrients, and electron donors/acceptors in the system. This cluster is the fundamental research theme of the whole field, and the findings lay a theoretical foundation for understanding the remediation mechanism of EPSs in complex environmental systems.

Cluster 3 (blue): Interactions between EPSs and pollutants and removal mechanisms

This cluster includes core keywords such as “heavy metals”, “antibiotics”, “microplastics”, “antibiotic resistance genes (ARGs)”, “adsorption”, “complexation”, “biodegradation”, “electron transfer”, “toxicity”. It is the core applied research cluster of the field and corresponds to the direct functional mechanism of EPSs in pollutant remediation: contaminant immobilization, transformation and degradation, and ecological risk regulation. The keywords in this cluster directly reflect the multiple pathways of EPSs in the remediation of different pollutants: through physical adsorption, chemical complexation, and ion exchange to achieve the immobilization of pollutants; through mediating electron transfer and providing active sites for extracellular enzymes to promote the degradation and transformation of pollutants; and through regulating the migration and horizontal gene transfer of ARGs to affect the ecological risk of emerging pollutants. This cluster has the most abundant keyword types and the fastest growth rate of occurrence frequency and is the core research hotspot of the current field.

Cluster 4 (red): Composition, structure and analytical methods of EPSs

This cluster focuses on keywords such as “protein”, “polysaccharide”, “extraction”, “characterization”, “fluorescence quenching”, “size exclusion chromatography”, “FTIR”, “molecular structure”, “functional groups”. It corresponds to the methodological foundation of the whole field: the structural characterization and component resolution of EPSs. The functional diversity of EPSs in bioremediation is essentially determined by their complex composition and spatial structure. Studies in this cluster are committed to developing efficient and non-destructive extraction methods and high-resolution characterization techniques for EPSs to clarify the relationship between the composition, functional groups, molecular structure of EPSs and their remediation function. This cluster is the earliest emerging basic research theme in the field and provides methodological support for all other research dimensions.

#### 3.5.2. Keyword Burst Analysis for EPS Research Hotspots

To further identify the evolutionary trends of research hotspots in microbial EPS bioremediation, keyword burst detection was performed using CiteSpace (6.1.R6) software. The top 20 keywords with the strongest burst strength are summarized in Table 3. Burst strength reflects the growth rate of keyword occurrences in a specific time window; a higher value indicates that the keyword has received rapidly increasing research attention, with more significant characteristics of an emerging research hotspot.

Early burst terms “extraction” (2010–2016) and “soluble microbial product” (2008–2016) indicated that early research focused on establishing methodological foundations, centering on the development of non-destructive extraction techniques and compositional characterization of EPSs [8]. Burst keywords such as “wastewater treatment” (2014–2020) and “heavy metals” (2015–2021) reflected a shift in research focus to engineering applications, exploring the functional roles of EPSs in wastewater treatment systems and their remediation potential for conventional pollutants [13]. For example, studies confirmed that EPSs were the core medium for activated sludge flocculation and heavy metal adsorption [12]. Recent burst keywords including “antibiotics” (2021–2025), “microplastics” (2022–2025), and “electron transfer” (2022–2025) highlight the current shift to emerging pollutant remediation and molecular mechanism elucidation, with particular attention to EPS interactions with microplastics/antibiotics and electron transfer processes. For instance, aromatic proteins in EPSs have been found to significantly promote microplastics aggregation [38]. The temporal variation of these burst keywords clearly demonstrates that EPS bioremediation research has evolved from methodological establishment to conventional application, and now to frontier exploration of emerging pollutants. The detailed evolutionary relationships between research themes are further analyzed in Section 3.6.

### 3.6. Thematic Evolution Analysis

**Figure 8:**
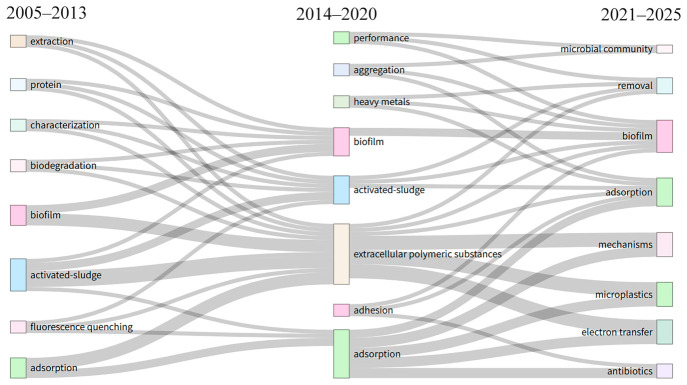
Thematic evolution Sankey diagram of EPS bioremediation research from 2005 to 2025.

To intuitively show the transformation of research themes over time, we divided the entire study period into three stages consistent with the publication trend analysis (2005–2013, 2014–2020, 2021–2025) and constructed a thematic evolution Sankey diagram (Figure 8) based on keyword clustering results.

Early research (2005–2013) centered on foundational methodology, with core themes focusing on extraction, characterization, protein, and biofilm in laboratory-controlled environmental systems. During this stage, research prioritized the development of extraction methods and structural characterization of EPSs, laying a fundamental framework for understanding their basic properties in simple laboratory settings. In contrast, the 2014–2020 period witnessed a transition toward conventional pollutant remediation and system performance optimization, with research hotspots shifting to heavy metals adsorption, activated sludge, and aggregation regulation. This phase marked a shift from basic laboratory research to practical application scenarios, where EPSs’ adsorption capacity for traditional pollutants and regulatory roles in wastewater treatment systems became the dominant focus. The most recent stage (2021–2025) reflects a remarkable thematic diversification and frontier breakthrough, with emerging core themes including microplastics, antibiotics, and electron transfer. This evolutionary trajectory highlights the expansion of the EPS research agenda, from basic property characterization and laboratory-scale exploration to interdisciplinary, multi-scenario bioremediation applications.

The shift from “conventional pollutant remediation” to “emerging pollutant control coupled with in-depth mechanistic exploration” has driven research to evolve from focusing on single pollutant removal efficiency to systematically dissecting the multi-interface interactions among pollutants, microorganisms, and EPSs; the rise of related themes such as mechanisms and microbial communities further reflects the shift of research focus to molecular-level action processes and complex microbial interaction networks. These findings indicate that microbial EPS bioremediation research has matured into a dynamic field, increasingly aligning with global environmental governance demands and practical applications in complex aquatic and soil ecosystems.

## 4. Current Research Progress and Hotspots

**Figure 9:**
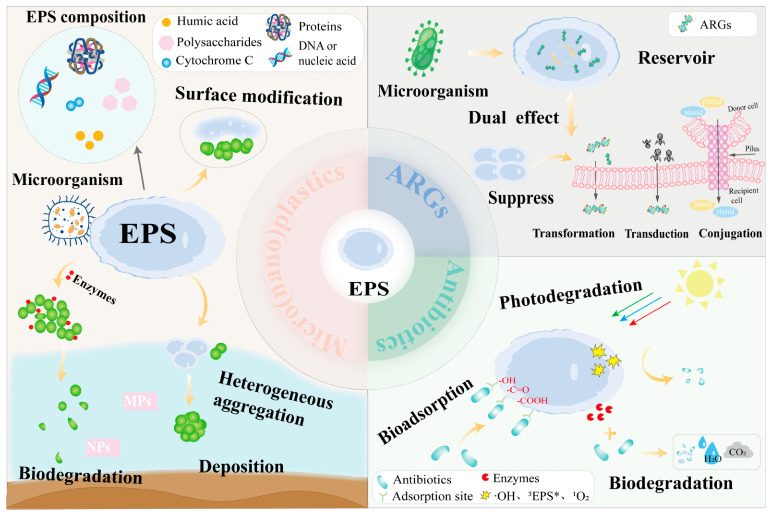
The interaction mechanisms between EPSs and micro(nano)plastics, antibiotics, and ARGs. Note: The * represents the excited state.

Based on keyword co-occurrence clustering, burst analysis (Section 3.5), and thematic evolution analysis (Section 3.6), the keyword cluster “interactions between EPS and pollutants and removal mechanisms” (Cluster 3) and the highest burst strengths for “antibiotics” (5.34), “microplastics” (4.87), “removal” (4.64) and “mechanism” (4.17) (Table 3) together pinpoint that the interaction mechanisms between EPSs and emerging pollutants constitute the most prominent research hotspot. Moreover, the functional mechanisms by which EPSs act as electroactive mediators in driving pollutant transformation and facilitating electron transfer had also been thoroughly discussed (burst strength: 2.99, continuous burst from 2022 to 2025). This section systematically reviews the progress of the above core hotspots, with the corresponding mechanistic pathways summarized in Figure 9. It should be noted that most studies reviewed in this section employed total EPS extracts without distinguishing S-EPSs, LB-EPSs, and TB-EPSs. The described mechanisms therefore reflect the net function of bulk EPSs and may oversimplify the often heterogeneous roles of individual fractions in real environmental systems.

### 4.1. Interaction Mechanisms Between EPSs and Emerging Pollutants

This section corresponds to Cluster 3 (interactions between EPSs and pollutants and removal mechanisms) in the keyword co-occurrence analysis. The following subsections elaborate on how EPSs interact with micro(nano)plastics, antibiotics, and ARGs, respectively.

Emerging pollutants are characterized by high toxicity, persistent resistance to degradation, and widespread mobility. Their accumulation in the environment not only threatens ecosystem stability but may also pose risks to human health through the food chain. Combining bibliometric findings (Section 3.4 and Section 3.5) with accumulating experimental evidence, it is now widely recognized that EPSs, as key reactive components in environmental matrices, exert profound influences on the environmental fate and ecological risks of emerging pollutants through multiple mechanisms including physical adsorption, chemical binding, and biological transformation. Table 4 shows the application of EPSs in micro(nano)plastics, antibiotics, and ARGs.

#### 4.1.1. Interactions Between EPSs and Micro(nano)plastics

Microplastics (MPs, <5 mm) and nanoplastics (NPs, <100 nm) have emerged as globally concerning environmental pollutants due to their potential biotoxicity and threats to ecosystems [49]. The interactions between EPSs and MPs/NPs exert significant effects on the ecological environment. As ubiquitous natural polymers in microbial communities and biofilm, EPSs mediate the aggregation, settling, surface modification, and adsorption of co-pollutants with MPs/NPs through their distinctive physicochemical properties, thereby regulating the environmental fate and bioavailability of these particles [38].

After MPs/NPs enter aquatic or soil environments, microorganisms tend to colonize their surfaces and secrete EPSs to form biofilm. Components of EPSs, such as polysaccharides, proteins, nucleic acids, and lipids, can bind to MPs/NPs through various mechanisms including hydrophobic interactions, electrostatic attraction, hydrogen bonding, and polymer bridging. From a molecular perspective, proteins are the most functionally active components within EPSs. Aromatic proteins, particularly those rich in tryptophan and tyrosine residues, serve as core mediators of hydrophobic interactions. The inherent benzene-ring structures within these proteins facilitate the adsorption of MPs like polyethylene (PE), thereby promoting particle aggregation [50]. This aligns with findings by Li et al., who reported that *C. sorokiniana* exposed to 3 µm polystyrene secreted increased amounts of aromatic protein-like substances, which enhanced hydrophobic interactions and aggregation between cells and MPs [51]. Previous studies have shown that the viscoelastic and hydrophobic nature of EPSs enables interactions with MPs/NPs, forming hetero-aggregation that reduce particle suspension and accelerate settling [52,53]. This process ultimately facilitates the separation and removal of MPs/NPs from aqueous environment. Rodrigues et al. found that the S-EPSs secreted by *Cyanocohnella rudolphia* under exposure to polystyrene microplastics (PS MPs) could induce hetero-aggregation of high-concentration PS MPs (2 g/L) through charge neutralization and bridging, achieving a removal rate of 82% [40]. In contrast, Cheng and Wang demonstrated that the B-EPSs from *Scenedesmus abundans* were more effective than S-EPSs in mediating the removal of PS, polymethyl methacrylate (PMMA) and polylactide (PLA) MPs, with overall removal exceeding 84% [39]. This discrepancy likely stems from inherent functional specificity of distinct EPS fractions, while the physicochemical properties and types of MPs further determine the dominant role of each fraction in hetero-aggregation. Unfortunately, most of the published studies still use unfractionated bulk EPS extracts, making it impossible to disentangle the specific contributions of individual fractions and leading to conflicting mechanistic interpretations. Furthermore, the high removal efficiencies reported in laboratory studies are rarely achievable in natural environments. Most aggregation experiments are conducted under optimized conditions (controlled pH, low ionic strength, single MP type and size) that do not reflect the complexity of real aquatic systems. EPSs not only facilitate the formation of hetero-aggregation between microbes and MPs but also enhance the interfacial adsorption of MPs onto aquatic sediments and soil particles, thereby further reducing the abundance of freely suspended MPs [39]. However, such aggregation and sedimentation do not constitute genuine degradative removal. In this process, EPSs function largely as “physical vectors,” merely facilitating the transfer of MPs/NPs from the water column to the underlying sediment. MPs/NPs enmeshed within EPSs are not completely mineralized upon settling. Instead, they tend to accumulate and persist within sediments or soil matrices, effectively transforming these depositional environments into secondary sources of pollution with the potential for future release.

MPs can adsorb metal ions and organic pollutants through electrostatic interactions, hydrophobic interactions, and complexation [54]. However, EPS coating significantly alters the surface properties of MPs/NPs (such as charge, hydrophobicity, and functional group composition), affecting their mobility and adsorption behavior toward other contaminants. Wang et al. reported that EPS-based biofilms formed in environmental media could significantly enhance the adsorption capacity of PE MPs for Cu(II), reaching 318 µg/g, far exceeding that of pristine microplastics (31.2 µg/g) [55]. This enhancement is primarily attributed to the abundant functional groups in EPSs (such as carboxyl, hydroxyl, and amide groups), which concentrate metal ions through complexation, ion exchange, and surface precipitation. However, this study is limited to single polymer types (e.g., PE) and short-term artificially cultured biofilms, lacking validation of long-term naturally mature biofilms in real environmental matrices. Laboratory-grown biofilms typically have thinner and more uniform EPS layers than natural biofilms, which develop over months to years and contain complex microbial communities. This makes direct comparison of adsorption capacities across studies extremely challenging. Furthermore, it only characterizes the glycosyl composition of EPSs, neglecting the contributions of critical components including proteins and nucleic acids to the adsorption behavior. Similarly, for organic pollutants, EPS-modified MPs often exhibit higher adsorption potential due to their increased specific surface area and enhanced surface affinity. Previous studies have shown that naturally aged microplastic fibers coated with biofilm (rich in EPSs) showed a 20–85% increase in adsorption capacity for perfluorooctane sulfonate (PFOS) compared to pristine samples [56]. However, this EPS-mediated enhancement of adsorption could cause EPS-MP complexes to act as chemical “Trojan horses” for hazardous environmental pollutants. Such a mechanism may amplify the enrichment of these pollutants in organisms and facilitate subsequent toxin release, potentially exacerbating ecological risks [57]. Current remediation research on MPs pollution remains largely focused on the removal of pristine MPs, while the synergistic ecological risks posed by EPS-MP complexes have yet to receive sufficient attention.

The microbial biodegradation of MPs/NPs is a complex biogeochemical process that typically involves three main stages: colonization, fragmentation, and assimilation [58]. The process begins with microbial attachment and biofilm formation on the plastic surface, establishing an active interface primarily composed of EPSs. EPSs not only provide physical protection and a stable microenvironment for microbial communities but also contain various enzymes (such as esterases, peroxidases, and oxygenases) that can directly cleave chemical bonds (e.g., ester bonds, C-C) in plastic polymer chains, initiating the initial degradation of the polymer [59]. Under the protection and catalytic influence of the biofilm, extracellular enzymes (such as esterases, lipases, and oxygenases) secreted by microorganisms can function efficiently. Various microorganisms, including bacteria, fungi, and algae, can degrade MPs/NPs. The majority of bacterial species with MPs/NPs degrading ability are affiliated with the phyla Proteobacteria, Firmicutes, and Actinobacteria [60]. For instance, colonization by *Bacillus cereus* led to a 10.7% mass loss of PS MPs after 50 days of incubation [61]. Fungi and algae also possess the capacity to degrade MPs. For instance, *Aspergillus flavus* degrades high-density PE MPs into lower molecular weight PE MPs after 28 days of incubation [62]. After 112 days, the microalga *Spirulina* sp. reduced the carbon content of polyethylene terephthalate (PET) and polypropylene (PP) MPs by 48.61% and 36.70%, respectively [63]. The degradation of MPs by diverse microorganisms is primarily dependent on the catalytic action of extracellular enzymes, which reveals the essential role of EPSs in the biodegradation of MPs/NPs.

Taking polystyrene (PS) as an example, its degradation is often initiated by oxygenases that catalyze the insertion of oxygen atoms into C-H bonds of the alkyl chain, forming hydroxyl (-OH) groups. These are subsequently oxidized to carbonyl (-C=O) and carboxyl (-COOH) groups, progressively increasing the hydrophilicity of the plastic surface and facilitating further enzymatic breakdown [64]. These reactions progressively break down long-chain polymers into oligomers, dimers, or monomers. Ultimately, the resulting small-molecule products can be assimilated by microorganisms and enter intracellular metabolic pathways (such as β-oxidation). These products are partly converted into cellular biomass and partly mineralized to carbon dioxide and water, thereby completing the transformation of plastics into bioavailable forms [65]. Throughout this process, EPSs serve not only as “enzyme reaction carriers” that maintain enzyme activity and enhance interfacial contact, but also regulate local chemical conditions and enrich degrading microbial populations, thereby synergistically advancing the biodegradation of MPs/NPs. Nevertheless, the efficiency of EPS-mediated biodegradation for MPs/NPs remains extremely low in natural environments. Current research is often constrained by an idealized approach: most laboratory studies employ purified EPSs, single efficient-degrading microbial strains, and pristine plastic particles. This approach largely overlooks real limiting factors such as plastic aging and modification, interference from co-existing pollutants, and competitive or antagonistic interactions within microbial communities. Consequently, degradation rates measured under laboratory conditions are typically far higher than those observed in nature, failing to reflect actual degradation efficiency [66]. Moreover, the gradual fragmentation of plastics induced by EPSs inevitably generates substantial quantities of submicron- and nano-sized plastic debris. These smaller particles exhibit enhanced mobility and greater biological toxicity, which may further amplify ecological risks. This issue represents a core concern urgently requiring attention in current research on microplastic degradation.

#### 4.1.2. Interactions Between EPSs and Antibiotics

The abuse and continuous discharge of antibiotics have become a global environmental and health crisis, posing not only direct ecotoxicity but also serious threats to public health security [67]. The primary mechanisms of antibiotic removal by microbial EPSs are bioadsorption and biodegradation.

EPSs adsorb antibiotics mainly via electrostatic interactions, hydrogen bonding, hydrophobic effects, and van der Waals forces, thereby facilitating their subsequent degradation [68]. Studies have demonstrated that protein components within EPSs play a dominant role in the adsorption process. Relevant typical examples are detailed in Table 4. Similarly, Wu et al. reported a positive correlation between protein content in EPSs from the *Chlorella pyrenoidosa* (FACHB-9) and the degradation efficiency of enrofloxacin (EFX) and ciprofloxacin (CFX), further underscoring the dominant role of proteins in EPS–antibiotic interactions [69]. Moreover, the secondary structure of proteins within EPSs underwent significant alterations under antibiotic stress. Specifically, the α-helix/(β-sheet + random coil) ratio and β-sheet content increased progressively with rising antibiotic concentrations [70]. However, the findings outlined above are largely derived from studies conducted under pure culture conditions in laboratory settings, using single antibiotics and controlled environmental parameters. The efficacy of EPS-mediated antibiotic removal in authentic wastewater environments, where multiple antibiotics, heavy metals, and dissolved organic matter coexist and compete for adsorption sites, remains largely unvalidated. Therefore, to advance the practical application of EPS-based technologies, further research is required to assess their efficacy against antibiotics in authentic wastewater environments.

The surfaces of EPSs are rich in functional groups such as hydroxyl, carboxyl, and amino groups, which can serve as binding sites for the specific adsorption of antibiotics [71,72]. For example, oxytetracycline (OTC) has been shown to bind to EPSs through C–H, C–O, –COOH, and N–H bonds [46]. Furthermore, both EPS and microbial cell surfaces generally carry a negative charge, enabling effective adsorption of positively charged antibiotics (e.g., tetracyclines (TC)) and thereby enhancing removal efficiency. The adsorption of antibiotics by microbial EPSs is influenced by multiple environmental factors, including antibiotic concentration, pH, temperature, and carbon source type [73]. Tan et al. demonstrated that carbon sources can significantly affect the adsorption efficiency of TC by altering the yield and composition (such as the protein/polysaccharide ratio) of EPSs produced by *Pseudomonas* sp. TC952. When 10 g/L peptone was used as the carbon source, *Pseudomonas* sp. TC952 achieved an efficient TC removal rate for 72.8% within 6 days. EPSs form a protective area to bioadsorb TC for reducing or preventing TC from entering the bacteria cells [74]. In addition to compositional changes, EPS types also exhibit significant differences under antibiotic stress. For instance, in their investigation into the effects of OTC on aerobic granular sludge, He et al. observed that exposure to a low concentration (50 μg/L) did not significantly alter the total EPS content, yet it triggered a transformation from TB-EPS to LB-EPS. In contrast, under high concentration exposure (5 mg/L), the TB-EPS increased markedly, suggesting that its dense structure plays a crucial role in defending against antibiotic toxicity [75]. Such dynamic changes in different EPS fractions under varying antibiotic concentrations demonstrate that the EPS matrix is not a homogeneous structure but has distinct functional layers. Collectively, EPSs not only promote the immobilization and enrichment of antibiotics but also create favorable conditions for subsequent biodegradation. On one hand, EPSs reduce the direct toxicity of antibiotics to microbial cells and provide a stable microenvironment. On the other hand, functional microorganisms embedded within or adsorbed onto the EPS matrix can secrete various extracellular degradative enzymes, such as oxidoreductases and hydrolases, to break down antibiotics into simpler molecules [76].

Moreover, EPSs can act as photosensitizers to promote antibiotic degradation in photocatalytic processes. Based on existing studies, EPS-mediated photodegradation primarily involves two mechanisms: (1) direct photolysis, where EPSs absorb photons to form excited states that induce structural changes such as aromatic ring cleavage and double-bond scission; (2) indirect photolysis, where reactive species including hydroxyl radicals (·OH), superoxide radicals (·O_2_^−^), singlet oxygen (^1^O_2_), hydrogen peroxide (H_2_O_2_), excited triplet-state EPS (^3^EPS*), and hydrated electrons (eₐq^−^) are generated under irradiation, thereby driving degradation reactions [45]. EPSs were derived from *Chlorella vulgaris* with high antibiotic photodegradation capability, and the removal efficiency of TC reached 56% at 0.6 V by integrating photosynthetic electron extraction and antibiotic induction. The protein and humic acid that are considered two main photoactive substances in EPSs produced at 0.6 V accumulated to a high level of 320 and 24 μg/cm^3^ and were further increased to 380 and 48 μg/cm^3^ when TC was added, which were 4.7 and 6.4-folds higher than that produced at potential free in the absence of TC [77]. It is noteworthy that the source and composition of EPSs significantly influence their photochemical behavior and degradation efficiency. Studies by Li et al. and He et al. consistently found that among the three typical EPS fractions (S-EPSs, LB-EPSs, and TB-EPSs), S-EPSs exhibit the highest photocatalytic activity, with significantly better degradation efficiencies for sodium dodecyl benzene sulfonate (SDBS) and TC than LB-EPSs and TB-EPSs. However, Li et al. attributed the superior performance of biofilm-derived S-EPSs to their lower molecular weight and aromaticity, which are more favorable for the generation of reactive intermediates (RIs); in contrast, He et al. found that the high photoactivity of sludge-derived S-EPSs is precisely due to its higher aromaticity, unsaturation, and lignin/CRAM component content [78,79]. S-EPSs are rich in redox-active compounds, including lignin-like substances and tannin-like components. These molecules contain abundant functional groups, such as phenolic hydroxyl, carboxyl, and methoxy moieties. Moreover, they possess dual characteristics as both efficient electron donors and acceptors. Under light irradiation, this enables rapid ground-to-excited state cycling, leading to the sustained and stable generation of ^3^EPS* and ^1^O_2_ as the dominant reactive species [45]. This photochemical process concurrently minimizes the quenching losses of these reactive species, ultimately achieving efficient and sustained photocatalytic degradation of recalcitrant pollutants such as antibiotics.

Despite the significant potential of EPSs for photocatalytic antibiotic degradation, their practical application faces several challenges. First, the environmental longevity of photosensitive components remains a concern: the photobleaching of these moieties under continuous irradiation leads to a gradual decline in catalytic activity. Second, there are potential environmental risks, as the photodegradation process may generate intermediate products with enhanced toxicity. Furthermore, current understanding of the underlying molecular regulatory mechanisms remains limited; key aspects such as electron transfer pathways and the expression of critical genes are still poorly defined. Future research should therefore focus on elucidating these mechanisms to provide a theoretical foundation for the development of highly efficient photocatalytic EPS systems.

#### 4.1.3. Interactions Between EPSs and ARGs

The overuse and improper disposal of antibiotics have led to their persistent presence in the environment, exerting long-term selective pressure that induces and promotes the emergence and spread of ARGs [80]. ARGs can be disseminated through two pathways: vertical gene transfer and horizontal gene transfer (HGT). Among these, HGT (including conjugation, transformation, and transduction) serves as the primary mechanism for ARG spread, often mediated by mobile genetic elements such as plasmids, transposons, and integrons [81,82]. Previous studies have indicated that EPSs play a dual role in ARG transfer: they may act as a reservoir for ARGs, yet can also suppress their dissemination [83]. For example, EPSs secreted by *Geobacter* primarily reduce HGT of ARGs by alleviating the oxidative stress induced by roxithromycin (ROX). Compared to conditions without EPSs, the transformation frequency of pBBR1MCS-3 was reduced by 66.8% in the presence of EPSs. Increased EPS content led to downregulation of *rpoS*, soxR, and *oxyR*. Upon removal of EPS protection, there was an elevation in the expression levels of *lexA*, *recA*, and *umuD* genes (bacterial SOS response) by 2.7-fold, 1.8-fold, and 2.8-fold, respectively. However, supplementation with EPSs and c-Cyts resulted in a decrease in gene expression associated with SOS response [48]. At the molecular level, carboxyl and amino groups of proteins like c-type cytochromes in EPSs interact with the phosphate backbone of ARGs via hydrogen bonding and impede ARG migration [48]. Meanwhile, residues including tryptophan and tyrosine also effectively bind ARGs, restricting their dissemination among hosts [84]. Furthermore, Liu et al. observed that increased EPSs in electrode biofilms reduced the abundance of ARGs in the anode region and influenced ARG distribution in microbial electrolysis cells [84].

However, certain components of EPSs may also facilitate ARG transfer. For instance, carbohydrates and aliphatic substances in S-EPSs and LB-EPSs can promote ARG dissemination by influencing the formation of reactive oxygen species (ROS) in S-EPSs and the production of both ROS and adenosine triphosphate (ATP) in LB-EPSs [85]. Increased EPS secretion and enhanced hydrophobic functional groups promote biofilm formation, thereby creating an environment conducive to the proliferation of ARGs [86]. Li et al. found that during the cultivation of soil microorganisms exposed to TC, elevated EPS secretion led to a significant increase in HGT of ARGs [87]. However, most of these findings originate from laboratory-scale experiments, including both pure culture systems (e.g., *Escherichia coli* or *Geobacter*) and simplified microcosm experiments using mixed microbial consortia. Although the study by Li et al. has advanced the application of environmentally derived microbial communities, direct evidence from complex in situ environments (e.g., natural soils, rivers, or sediments) remains very limited. Therefore, the discussion on EPSs facilitating ARG transfer via HGT requires further validation under environmentally relevant conditions. Moreover, under pollutant stress (e.g., antibiotics or heavy metals), stress-induced microbial EPS secretion may further exacerbate ARG dissemination risks. For instance, trace antibiotics in systems like sewage pipelines can modulate EPS composition and microbial community structure, thereby increasing the potential for ARG spread [88].

Notably, EPSs are not homogeneous substances. Their distinct layers, such as LB-EPSs and TB-EPSs, exhibit marked structural and functional differences. These variations directly dictate the complexity of their interactions with ARGs. Studies have demonstrated that differences in the concentration and characteristics of LB-EPSs and TB-EPSs significantly influence their adsorption behavior toward pollutants [89]. Higher concentrations of TB-EPSs may absorb ARGs more effectively, likely due to the greater sensitivity of TB-EPSs to changes in the external environment. Moreover, the dense structure of TB-EPSs can immobilize functional enzymes (e.g., nucleases like DNase I or oxidases like laccase), thereby preserving their activity for sustained degradation of free ARGs (e.g., cleaving blaTEM-1 gene fragments) [90]. In contrast, carbohydrates and aliphatic substances within S-EPSs and LB-EPSs may facilitate the horizontal transfer of ARGs, potentially by influencing the formation of reactive oxygen species ROS and ATP synthesis. This “functional stratification” reveals the structural basis for the dual role of EPSs in regulating ARG dissemination: the TB-EPS layer tends to “immobilize and degrade” ARGs, whereas the LB-EPS and S-EPS layers are more inclined to “facilitate and propagate” ARGs. Therefore, future research should shift its focus from “bulk EPSs” to “EPS fractions,” systematically comparing the relative contributions of different EPS layers and their subcomponents to ARGs adsorption, degradation, and transfer.

### 4.2. The Role of EPSs in Electron Transfer

Keyword burst analysis shows that “electron transfer” has a burst strength of 2.99 (Table 3), with a continuous strong research burst from 2022 to 2025, and is also a core keyword in Cluster 3. Based on Section 3.5, Section 3.6, and a critical review of experimental studies, it is reasonable to conclude that “electron transfer” serves as a core mechanism through which EPSs mediate pollutant transformation, offering an important pathway for the treatment of emerging contaminants.

EPSs are rich in substances with redox activity and can act as a transient mediator in the process of microbe-mediated extracellular electron transfer (EET). The electroactivity of EPSs primarily originates from redox-active components such as proteins, c-type cytochromes, flavin-like compounds, and humic acids [91]. These components effectively mediate EET via electron hopping or electron shuttling mechanisms, reducing charge-transfer resistance and reaction activation energy, thereby accelerating electron exchange between microorganisms and extracellular electron donors or acceptors [92]. For example, EPSs secreted by electroactive bacteria, such as *Shewanella oneidensis* MR-1, have been shown to directly participate in microbial EET. These bacteria are capable of exchanging electrons with solid materials, including electrodes and metal oxides. In this process, EPSs can act as a transient medium through which electrons are transferred via an electron-hopping mechanism [93]. In a study by Zhou et al., EPSs extracted from *Brucella intermedia* ZL-06 functioned as electron shuttles, enhancing the degradation efficiency of AZI by white-rot fungi by 33.3% through the promotion of ROS generation, strengthened EET, and upregulation of key enzyme activities such as laccase [44]. However, the relatively low efficiency of electron transfer between electroactive microorganisms and their electron acceptors remains a critical bottleneck in the practical application of EET. Electric fields have been found to enhance EET and the application of an electric field to *Geobacter sulfurreducens* biofilms has been shown to stimulate the production of cytochrome *OmcZ* nanowires, whose conductivity is 1000-fold higher than that of cytochrome *OmcS* nanowires [92]. Jiang et al. reported that electrical stimulation increased the conductivity and capacitance of EPSs by 1.27–1.32-fold and 1.32–1.83-fold, respectively [94].

Generally, the electron transfer rate in LB-EPSs exhibits a relative advantage. The protein content of LB-EPSs is relatively higher than that of TB-EPSs. During electron transfer, proteins demonstrate higher conductivity than polysaccharides, while components such as c-type cytochromes and thiol-containing proteins directly participate in redox reactions, storing and transferring electrons to form efficient electron transfer pathways [95,96]. Moreover, functional groups in EPSs, including carbonyl and hydroxyl groups, also exhibit redox activity and can serve as natural electron carriers, enhancing the overall activity of the electron transfer system [97]. Zhu et al. investigated changes in the EPSs of *G. sulfurreducens* biofilms under low concentrations of TC (μg/L to mg/L) and further elucidated the impact of these EPS changes on TC resistance through transcriptomic analysis. The results indicated that low concentrations of TC altered both the structure and composition of EPSs. In this study, 0.05 mg/L TC upregulated the expression of most genes directly associated with EET (including *nuoI-1*, *nuoG-1*, *nuoL-1*, *nuoN-1*, *nuoM-1*, *omcO*, *omcB*, *omcM*, *pilA-C*, *pilA-N* and *pilR*), thereby enhancing the efficiency of EET [98].

In denitrification processes, the addition of EPSs significantly improves denitrification performance. For instance, Wang et al. demonstrated that EPSs extracted from waste-activated sludge increased the nitrate reduction rate by approximately 1.42-fold while lowering charge transfer resistance and elevating the activities of nitrate reductase and nitrite reductase [99]. Moreover, EPSs promoted carbon source metabolism and stimulated the production of coenzymes such as nicotinamide adenine dinucleotide (NADH), thereby supplying additional reducing power for denitrification. Acting similarly to quinones in electron transfer chains, EPSs effectively accelerated electron flow to nitrate or nitrite [100]. Similarly, in anammox systems, the addition of EPSs also significantly enhanced the activity of anammox bacteria, increasing ammonia and nitrite removal rates by 1.53-fold and 1.88-fold, respectively. These findings further confirm the role of EPSs as electron bridges in nitrogen transformation [101].

In heavy metal remediation, the electroactive properties of EPSs can promote the reduction and immobilization of metal ions. Redox-active components within EPSs reduce high-valent heavy metal ions, such as Cr(VI) and As(V), to lower-valent states [102,103]. For example, EPSs secreted by *Shewanella oneidensis* MR-1 can directly mediate the reduction of U(VI) to U(IV) via c-type cytochromes [104]. In chromium remediation, EPSs not only act as electron shuttles to accelerate microbial reduction of Cr(VI) to Cr(III) but also immobilize the generated Cr(III) within the EPS matrix through adsorption, achieving efficient heavy metal removal [105]. Although the mechanisms of EPSs in heavy metal remediation have been widely studied, future work in this field still requires greater integration of technological innovation, multi-scenario applications, and engineering practices.

## 5. Challenges and Future Perspectives of Microbial EPS Engineering Applications

Despite the promising potential of microbial EPSs in bioremediation, their widespread implementation in large-scale engineering systems remains constrained by several key challenges. Based on bibliometric analysis and recent research advances, this section systematically outlines the major obstacles to the scalable application of EPSs and discusses possible future development directions.

### 5.1. Main Challenges in Engineering Application

Bibliometric analysis shows that although the number of publications in this field has grown rapidly (especially after 2021), EPS bioremediation research remains at the stage of laboratory-scale mechanistic exploration, and large-scale engineering applications are still constrained by three core challenges.

First, the high cost and low yield of EPSs represent the most fundamental challenge restricting its engineering application. Currently, most studies employ purified EPSs obtained from laboratory-scale pure culture strains. The large-scale production of EPSs relies on microbial fermentation technologies. However, widespread application is hindered by generally low yield, complex extraction processes, and high energy consumption, which collectively compromise economic feasibility in practical engineering. In particular, scalable separation and purification technologies for high-purity EPSs remain underdeveloped, which limits their industrial-scale application. Li et al. reported that the EPS yield from activated sludge constitutes 9–19% of the total volatile solids, which is significantly lower than that from granular sludge, where the yield ranges from approximately 19% to 30% [106]. Additionally, EPS synthesis is influenced by multiple factors, including microbial strain variations, culture conditions, and environmental stressors. These variables can lead to considerable fluctuations in the chemical composition and physicochemical properties of EPSs, thereby significantly compromising the stability and predictability of their remediation performance. Achieving standardized EPS functionality through process optimization thus remains a critical challenge for practical applications.

Second, the mechanisms by which EPSs function under complex environmental conditions remain poorly understood. Although existing studies have elucidated the multifaceted roles of EPSs in adsorption, complexation, electron transfer, and photocatalytic degradation, most findings are derived from single train or pure culture systems. Consequently, they fail to accurately reflect the behavioral pathways of EPSs in real environments characterized by coexisting pollutants and multi-media interactions. For instance, while EPSs can mediate the hetero-aggregation and sedimentation of MPs, the secondary NPs generated during this process and their associated ecological risks have yet to be comprehensively assessed. Similarly, the “dual role” of EPSs in either inhibiting or facilitating the dissemination of ARGs lacks systematic validation, particularly regarding the poorly understood functional differentiation among EPS fractions such as S-EPSs, LB-EPSs, and TB-EPSs.

Furthermore, urgent attention must be directed toward the ecological risks and long-term stability of EPSs during remediation processes. As macromolecular biopolymers, EPSs themselves can be utilized by microorganisms in the environment, potentially triggering secondary pollution or altering pollutant migration pathways. For instance, EPS-MP complexes may act as chemical “Trojan horses,” exacerbating the enrichment of hazardous substances along the food chain. Similarly, in the process of photocatalytic antibiotic degradation, the photobleaching of EPSs and the associated toxicity changes of intermediate products remain systematically unevaluated. Current research predominantly focuses on short-term remediation efficiency, while insufficient attention has been paid to the long-term fate of EPSs in real environments, the toxicity of its degradation byproducts, and the corresponding ecological health risks. Therefore, future efforts should simultaneously advance the enhancement of production efficiency, the elucidation of complex mechanisms, and the refinement of risk assessment protocols to facilitate the transition of EPS-based technologies from laboratory-scale research to engineering applications.

### 5.2. Future Prospects and Research Directions

In response to the three core challenges outlined above, low production efficiency, poorly understood mechanisms in complex environments, and unclear ecological risks, future research should be advanced in the following directions.

#### 5.2.1. Waste Valorization

Driven by the bibliometric finding that “resource recovery” and “waste activated sludge” constitute a core keyword cluster (Cluster 1), efforts can be focused on resource recovery strategies based on waste biomass, so as to address the industrial bottlenecks of high production costs and low yield (Section 5.1). Two complementary approaches can be pursued. First, organic-rich wastes, such as waste-activated sludge, agricultural straw, and food processing wastewater, can serve as feedstocks to induce in situ EPS production by functional microorganisms through optimized fermentation conditions, achieving “waste treatment by waste.” For example, Ng et al. recovered mixed culture resources from industrial glycerin pitch (GP). In batch tests with 1.5 g C/L of GP, the enriched microorganisms removed 83% of the organic compounds and produced 128 mg EPSs per g volatile solids. The EPSs extracted displayed optimal kaolin flocculation activities of 100% at 2.5 mL dosage and pH 7 using 1% (*w*/*v*) calcium chloride, demonstrating their potential to replace synthetic flocculants under laboratory conditions [107]. Second, mild and efficient EPS extraction and purification techniques should be developed, including low-temperature thermal treatment, acid–alkali synergistic extraction, and membrane separation-coupled processes. These methods aim to preserve EPS bioactivity while reducing energy consumption and chemical usage. Furthermore, the recovered EPSs hold promise for diverse applications beyond environmental remediation. They can be utilized as bioflocculants or soil conditioner for environmental remediation. Additionally, they show promise as green scale inhibitors in industrial recirculating water systems and membrane separation processes, offering a sustainable alternative to traditional chemical scale inhibitors (e.g., phosphorus-containing compounds and synthetic organic polymers) and thereby reducing environmental burden.

#### 5.2.2. Synthetic Biology Regulation

To address the substantial compositional fluctuations and inconsistent performance of EPSs (Section 5.1), synthetic biology and metabolic engineering approaches should be employed to construct engineered strains with enhanced productivity and controllable functionality. Multi-omics techniques, including metagenomics, transcriptomics, and proteomics, can be applied to elucidate the biosynthetic pathways of EPSs in different functional microorganisms. This enables the identification of key gene modules and metabolic nodes that regulate the synthesis of EPS components such as aromatic proteins, c-type cytochromes, and redox-active functional groups. Furthermore, site-directed nucleases can be utilized in genome editing techniques to knock out genes or insert transgenes. Such engineering strategies allow for the selective enrichment of functional components involved in pollutant adsorption, electron transfer, and photocatalytic degradation, thereby enhancing the targeted remediation capacity of EPSs against emerging contaminants including microplastics, antibiotics, and heavy metals.

#### 5.2.3. Functional Resolution of EPS Fractions

Although preliminary evidence from corrosion studies demonstrates that S-EPSs, LB-EPSs, and TB-EPSs can exhibit contrasting functions, such fraction-level understanding remains virtually absent in the context of emerging pollutant remediation. As noted in Section 4, mechanistic claims regarding contaminant adsorption, degradation, and electron transfer are predominantly derived from total EPSs, leaving the distinct roles of individual fractions poorly understood. This knowledge gap is particularly evident in the dual role of EPSs in ARG dissemination. To bridge this gap, advanced characterization techniques, including excitation-emission matrix (EEM) fluorescence spectroscopy, Fourier transform infrared spectroscopy (FTIR), and X-ray photoelectron spectroscopy (XPS), should be employed to resolve the compositional and structural differences among the three fractions. Subsequently, isothermal titration calorimetry (ITC) and quartz crystal microbalance with dissipation monitoring (QCM-D) can quantify the binding kinetics (binding constants, binding energy, and adsorption rates) between each EPS fraction and pollutants such as microplastics, antibiotics, and ARGs. These data will enable the construction of a fraction-specific functional response model that elucidates the synergistic and antagonistic mechanisms governing pollutant adsorption, degradation, and horizontal gene transfer.

#### 5.2.4. Establishment of a Risk Assessment Framework

In light of the identified focus on emerging pollutants such as microplastics and antibiotics, future efforts should systematically evaluate the long-term fate and the currently overlooked ecological health risks of EPSs and their degradation products in real environments. Key priorities in this regard include the following. First, methods should be developed to trace the migration and transformation of EPSs in soils, aquatic systems, and organisms, along with the identification of degradation product composition and associated toxicity characteristics. Second, a multidimensional risk assessment framework should be constructed that encompasses bioaccumulation, the risk of ARG dissemination, and disturbances to microbial communities. Third, predictive models for the environmental behavior of EPSs should be developed by integrating machine learning and molecular simulation approaches. Such models will provide a scientific basis for establishing standards and risk control measures for engineering applications.

## 6. Conclusions

This study systematically reviewed the research landscape of microbial EPSs in bioremediation from 2005 to 2025 using bibliometric analysis. A steady increase in publication output was observed, with a notable acceleration after 2021, reflecting the growing research attention and perceived significance of EPSs in environmental remediation. While China led in the volume of published papers, there remains substantial potential to enhance international collaboration and produce higher-impact research. Future efforts should prioritize strengthening academic partnerships with leading research teams.

Through multifaceted interactions with emerging contaminants, EPSs exhibit a central regulatory function in bioremediation. They engage in hetero-aggregation and surface modification of microplastics, biosorption and photosensitized degradation of antibiotics, and exert dual regulatory effects on the dissemination of ARGs, operating via pathways such as physical adsorption, photocatalytic degradation, and EET. Notably, during electron transfer processes, the redox-active constituents of EPSs effectively mediate extracellular electron transport, substantially enhancing nitrogen removal efficiency and heavy metal reduction, thereby serving as a critical bridge connecting microbial metabolism with pollutant transformation.

Importantly, the functions of EPSs are not uniform. Their stratified architecture, comprising S-EPSs, LB-EPSs, and TB-EPSs, exhibits marked differences in composition, functionality, and environmental responsiveness, which collectively determine their diversified roles in pollutant fate. Despite the promising remediation performance of EPSs demonstrated at the laboratory scale, their large-scale application remains constrained by challenges including low production yield, high costs, complex environmental behavior, and unclear ecological risks.

Future efforts should be directed toward four strategic priorities: valorization of waste biomass for cost-effective EPS production, synthetic biology-enabled engineering of strains with tailored functionalities, systematic elucidation of the functional differentiation among EPS fractions, and establishment of a full lifecycle risk assessment framework. Advancing these directions may facilitate the translation of EPSs from fundamental research toward practical engineering applications.

## Figures and Tables

**Table 1:** Top 10 most productive authors in the field of microbial EPSs.

Rank	Authors	Documents	Citations	Citation Frequency per Document	Total Link Strength
1	Sheng, Guoping	16	4395	274.69	45
2	Yu, Hanqing	13	4496	345.85	36
3	Van Loosdrecht, Mark C. M.	12	820	68.33	28
4	Fang, Fang	10	921	92.10	33
5	Lin, Yuemei	10	515	51.50	28
6	Kang, Fuxing	7	519	74.14	8
7	Liu, Yang	7	273	39.00	7
8	Seo, Youngwoo	7	518	74.00	11
9	Zhang, Peng	7	512	73.14	23
10	Zhang, Wei	7	397	56.71	10

**Table 2:** Top 10 articles with the most citations for microbial EPS research.

First Author	Publication Year	Title	Journal Name	Total Citations	Reference
Sheng Guoping	2010	Extracellular polymeric substances (EPS) of microbial aggregates in biological wastewater treatment systems: A review	Biotechnology Advances	2515	[8]
Ohana Yonara De Assis Costa	2018	Microbial Extracellular Polymeric Substances: Ecological Function and Impact on Soil Aggregation	Frontiers in Microbiology	886	[26]
More T.T.	2014	Extracellular polymeric substances of bacteria and their potential environmental applications	Journal of Environmental Management	791	[27]
Hou Xiaolin	2015	Role of extracellular polymeric substance in determining the high aggregation ability of anammox sludge	Water Research	700	[30]
Xiao Rui	2016	Overview of microalgal extracellular polymeric substances (EPS) and their applications	Biotechnology Advances	664	[31]
Jia Fangxu	2017	Stratification of Extracellular Polymeric Substances (EPS) for Aggregated Anammox Microorganisms	Environmental Science & Technology	517	[32]
Alan W. Decho	2017	Microbial Extracellular Polymeric Substances (EPSs) in Ocean Systems	Frontiers in Microbiology	484	[33]
Yin Cuiqin	2015	Spectroscopic characterization of extracellular polymeric substances from a mixed culture dominated by ammonia-oxidizing bacteria	Water Research	470	[34]
Appala R. Badireddy	2010	Role of extracellular polymeric substances in bioflocculation of activated sludge microorganisms under glucose-controlled conditions	Water Research	441	[35]
Shi Yahui	2017	Exploiting extracellular polymeric substances (EPS) controlling strategies for performance enhancement of biological wastewater treatments: An overview	Chemosphere	415	[36]

**Table 3:** Top 20 keywords with the strongest citation bursts in EPS bioremediation research. Note: Red indicates the citation burst period of the keyword (corresponding to the Begin and End columns); Cyan indicates the non-burst research period.

Keywords	Year	Strength	Begin	End	2008–2025
soluble microbial product	2008	4.05	2008	2016	▃▃▃▃▃▃▃▃▃ ▂▂▂▂▂▂▂▂▂
extraction	2008	4.25	2010	2016	▂▂ ▃▃▃▃▃▃▃ ▂▂▂▂▂▂▂▂▂
matrix	2009	3.19	2013	2016	▂ ▂▂▂▂ ▃▃▃▃ ▂▂▂▂▂▂▂▂▂
wastewater treatment	2008	3.37	2014	2020	▂▂▂▂▂▂ ▃▃▃▃▃▃▃ ▂▂▂▂▂
heavy metals	2014	3.28	2015	2021	▂▂▂▂▂▂ ▂ ▃▃▃▃▃▃▃ ▂▂▂▂
water	2009	3.95	2020	2023	▂ ▂▂▂▂▂▂▂▂▂▂▂ ▃▃▃▃ ▂▂
removal	2008	4.64	2021	2023	▂▂▂▂▂▂▂▂▂▂▂▂▂ ▃▃▃ ▂▂
antibiotics	2020	5.34	2021	2025	▂▂▂▂▂▂▂▂▂▂▂▂ ▂ ▃▃▃▃▃
stability	2016	3.36	2021	2022	▂▂▂▂▂▂▂▂ ▂▂▂▂▂ ▃▃ ▂▂▂
nitrification	2021	2.73	2021	2022	▂▂▂▂▂▂▂▂▂▂▂▂▂ ▃▃ ▂▂▂
mechanism	2022	4.17	2022	2025	▂▂▂▂▂▂▂▂▂▂▂▂▂▂ ▃▃▃▃
electron transfer	2019	2.99	2022	2025	▂▂▂▂▂▂▂▂▂▂▂ ▂▂▂ ▃▃▃▃
microplastics	2013	4.87	2022	2025	▂▂▂▂▂ ▂▂▂▂▂▂▂▂▂ ▃▃▃▃
microbial community	2016	2.76	2022	2023	▂▂▂▂▂▂▂▂ ▂▂▂▂▂▂ ▃▃ ▂▂
role	2020	2.63	2022	2023	▂▂▂▂▂▂▂▂▂▂▂▂ ▂▂ ▃▃ ▂▂
nitrogen removal	2014	2.5	2022	2025	▂▂▂▂▂▂ ▂▂▂▂▂▂▂▂ ▃▃▃▃
scale inhibition	2022	2.33	2022	2022	▂▂▂▂▂▂▂▂▂▂▂▂▂▂ ▃ ▂▂▂
antibiotic resistance genes	2011	2.61	2023	2025	▂▂▂ ▂▂▂▂▂▂▂▂▂▂▂▂ ▃▃▃
nanoparticle	2012	2.6	2023	2023	▂▂▂▂ ▂▂▂▂▂▂▂▂▂▂▂ ▃ ▂▂
anammox	2018	2.38	2024	2025	▂▂▂▂▂▂▂▂▂▂ ▂▂▂▂▂▂ ▃▃

**Table 4:** Application of EPSs in removal of micro(nano)plastics, antibiotics and ARGs.

EPS Strain Used	Pollutants	Mechanism of Removal	Interaction	References
*Scenedesmus abundans*	Microplastics	Hetero-aggregations	MPs removal exceeded 84%. B-EPSs are more effective in inducing flocculation of MPs.	[39]
*Cyanocohnella rudophila*	Polystyrene MPs (2 g/L) (PS-MPs)	Charge neutralization/ bridging	Removal efficiency reached 82% under conditions of 0.05% Fe^3+^, pH 3.5, low salinity, S-EPS:PS-MPs ratio of 1:5, and reaction time of 60 min.	[40]
Activated sludge cultivated with NaAc, MeOH, and GLC as carbon sources	Polystyrene nanoplastics (PS-NPs)	Adsorption and flocculation	C=O stretching vibrations (amide I region) in EPS proteins preferentially bound to PS-NPs, increasing β-sheet content and enhancing protein rigidity to promote flocculation.	[41]
*Klebsiella* sp. J1	Sulfonamide antibiotics (SMX, SM1, SM2, SDZ)	Biosorption	Adsorption capacity of EPSs for SMX reached 70.0%. Tryptophan and tyrosine residues in EPS proteins mediated hydrophobic interactions with sulfonamide antibiotics.	[42]
Aerobic granular sludge (AGS)	Tetracycline (TC)	Biosorption	EPSs accounted for 40.9% of total TC removal. Protein (PN) content and PN/PS ratio positively correlated with sludge flocculation and granulation.	[43]
*Brucella intermedia* ZL-06	Azithromycin (AZI)	Biodegradation	EPSs enhanced ROS generation, increasing AZI degradation efficiency by 33.3% with a removal rate >99.9%.	[44]
	Tetracycline (TC)	Photosensitized degradation	TC removal by S-EPSs reached 48.3% in 5 h (k = 0.1363 h^−1^), outperforming B-EPSs (36.6%, k = 0.0902 h^−1^).	[45]
*Chlorella vulgaris* (FACHB-8)	Oxytetracycline (OTC)	Photodegradation	EPSs contain abundant tryptophan-like and protein-like substances, exhibiting high photosensitivity and facilitating triplet excited-state generation.	[46]
Aerobic granular sludge (AGS)	Sulfamethoxazole (SMX)	Biodegradation	SMX spontaneously bound to EPSs via enthalpy-driven interactions, primarily governed by hydrogen bonding and van der Waals forces.	[47]
*Geobacter*	ARGs (pBBR1MCS-3)	EPS/c-Cyts formed complexes with ARGs	Transformation frequency of pBBR1MCS-3 decreased by 66.8% in the presence of EPSs. Carboxyl, hydroxyl, and amide groups in EPSs and c-type cytochromes facilitated ARGs binding.	[48]

## Data Availability

The original contributions presented in this study are included in the article. Further inquiries can be directed to the corresponding author.
